# A Prokaryotic S1P Lyase Degrades Extracellular S1P *In Vitro* and *In Vivo*: Implication for Treating Hyperproliferative Disorders

**DOI:** 10.1371/journal.pone.0022436

**Published:** 2011-08-01

**Authors:** Andrea Huwiler, Florence Bourquin, Nataliya Kotelevets, Oleksandr Pastukhov, Guido Capitani, Markus G. Grütter, Uwe Zangemeister-Wittke

**Affiliations:** 1 Institute of Pharmacology, University of Bern, Bern, Switzerland; 2 Institute of Biochemistry, University of Zürich, Zürich, Switzerland; 3 Biomolecular Research, Paul Scherrer Institute, Villigen, Switzerland; Instituto Butantan, Brazil

## Abstract

Sphingosine-1-phosphate (S1P) regulates a broad spectrum of fundamental cellular processes like proliferation, death, migration and cytokine production. Therefore, elevated levels of S1P may be causal to various pathologic conditions including cancer, fibrosis, inflammation, autoimmune diseases and aberrant angiogenesis. Here we report that S1P lyase from the prokaryote Symbiobacterium thermophilum (StSPL) degrades extracellular S1P in vitro and in blood. Moreover, we investigated its effect on cellular responses typical of fibrosis, cancer and aberrant angiogenesis using renal mesangial cells, endothelial cells, breast (MCF-7) and colon (HCT 116) carcinoma cells as disease models. In all cell types, wild-type StSPL, but not an inactive mutant, disrupted MAPK phosphorylation stimulated by exogenous S1P. Functionally, disruption of S1P receptor signaling by S1P depletion inhibited proliferation and expression of connective tissue growth factor in mesangial cells, proliferation, migration and VEGF expression in carcinoma cells, and proliferation and migration of endothelial cells. Upon intravenous injection of StSPL in mice, plasma S1P levels rapidly declined by 70% within 1 h and then recovered to normal 6 h after injection. Using the chicken chorioallantoic membrane model we further demonstrate that also under *in vivo* conditions StSPL, but not the inactive mutant, inhibited tumor cell-induced angiogenesis as an S1P-dependent process. Our data demonstrate that recombinant StSPL is active under extracellular conditions and holds promise as a new enzyme therapeutic for diseases associated with increased levels of S1P and S1P receptor signaling.

## Introduction

Sphingolipids are essential constituents of cellular membranes and serve as signalling molecules involved in various physiological and pathophysiological processes. Sphingosine-1-phosphate (S1P) plays a key role in regulating cell proliferation and survival, cell migration, angiogenesis, as well as inflammatory processes and immune functions [1], [2], [3], [4], [5]. S1P is present in blood at high nanomolar concentrations due to the S1P-producing activity of sphingosine kinases (SK1) in various cell types including mast cells, erythrocytes and vascular endothelial cells [6], [7], [8], [9]. In blood S1P is bound to serum albumin and high density lipoproteins, which serve as buffers to decrease the pool of free S1P known to promote cardiovascular inflammation [10], [11], [12]. Interestingly, high levels of S1P are also generated by sphingosine kinases overexpressed in cancer cells, where it contributes to malignant progression and drug resistance as part of the sphingolipid rheostat counteracting pro-apoptotic sphingosine and ceramide [3], [13]. In addition to its intracellular function, secreted S1P may exacerbate disease progression by auto- and paracrine stimulation of S1P cell surface receptors [14], [15], [16]. So far, five receptor subtypes have been identified and denoted as S1P_1–5_ [17], [18], [19]. Their activation triggers downstream signaling via mitogen-activated protein kinases (MAPK), phosphoinositide 3-kinase, cyclic AMP and other mediators of cellular responses. Subsequent biological effects include cytoskeletal rearrangements, cell proliferation and migration, invasion, vascular development, platelet aggregation and lymphocyte trafficking [14], [20].

Although elevated S1P is causal or at least contributory to major human diseases, its cytoprotective effect is also important to maintain the function of normal vital tissues such as the immune and the cardiovascular system. To sustain controlled amounts of this highly bioactive lipid in tissues, S1P is irreversibly degraded by intracellular S1P lyase into hexadecenal and phosphoethanolamine. Decreasing the concentration of extracellular S1P or antagonizing S1P receptors may have therapeutic potential for various pathologic conditions including cancer, fibrosis, inflammation, autoimmune diseases, diabetic retinopathy and macular degeneration [3], [21], [22], [23], [24]. The sphingosine analogue FTY720 (fingolimod) is an immunosuppressive agent used for the treatment of multiple sclerosis and other autoimmune diseases [5], [25], [26]. Its *in vivo* phosphorylated form acts as an agonist on all S1P receptors, except S1P_2_. In addition, FTY720-phosphate may also indirectly antagonize S1P receptor signaling by receptor downregulation, thereby rendering cells unresponsive to S1P [5], [26], [27]. This ambivalent behaviour may result in unpredictable effects *in vivo*, therefore limiting the therapeutic use of this compound. As a more predictable approach, an anti-S1P antibody has recently been described, which acts as a molecular “sponge” to reduce the pool of endogenous circulating S1P [28]. However, it is questionable whether the reversible absorption of S1P with a neutralizing antibody can compete with the continuous release of S1P from blood and various other cell types.

A similar but likely more effective approach may be the use of S1P degrading enzymes like S1P lyase to irreversibly remove S1P from the circulation. S1P lyase has been cloned from various species including yeast [29], mouse [30], and human [31]. In mammalian cells, the enzyme is normally located intracellularly in the ER membrane with its active site facing the cytosol, and its main function may therefore be the degradation of intracellular S1P.

Recently, we have cloned and characterized the structure and function of S1P lyase from *Symbiobacterium thermophilum* (StSPL) [32]. In contrast to the enzymes from yeast, mouse and human, StSPL lacks a typical predicted transmembrane helix [32], and its structure solved at 2.0 Å resolution revealed that the active protein is a typical type I-fold dimeric pyridoxal-5′-phosphate (PLP)-dependent enzyme in which residues from both subunits contribute to the active site. The purified protein was able to cleave S1P in vitro [32].

Here, we demonstrate for the first time that recombinantly produced StSPL effectively degrades S1P in cell culture medium and in blood *in vitro* and *in vivo*. Using distinct cell types as *in vitro* models of cancer, fibrosis and aberrant angiogenesis, evidence is provided that StSPL disrupts S1P receptor signaling and thus mitigates pathophysiologic processes associated with increased levels of extracellular S1P. Furthermore, we used the chicken chorioallantoic membrane (CAM) as a neovascularization model to show the effect of StSPL on *in vivo* angiogenesis.

## Results

### Biochemical characterization of recombinant StSPL

**Figure 1:**
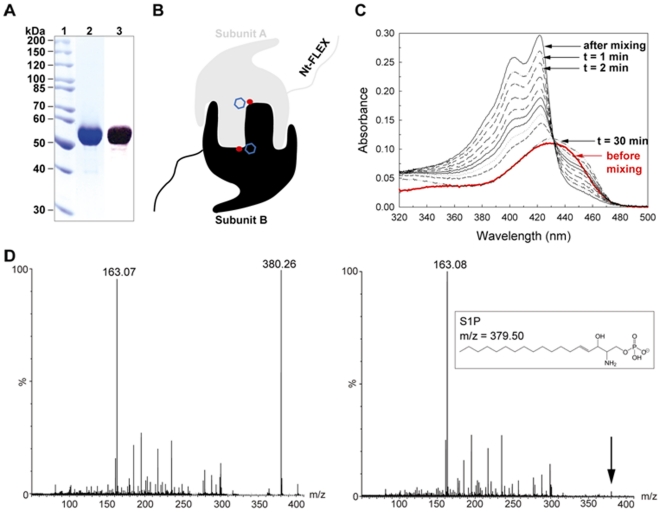
Biochemical characterization of StSPL. (A) Purity of purified WT StSPL. The molecular weight marker is shown in lane 1, the pooled fractions after size-exclusion chromatography were detected by Coomassie staining of the gel (lane 2) and by Western blotting with an antibody recognizing the C-terminal His-tag (lane 3). (B) Schematic representation of the StSPL dimer. Subunit A is depicted in grey, whereas subunit B is in black. A phosphate ion found in the active site of both subunits is depicted as a red dot, while the cofactor (PLP) is denoted by a blue hexagon. (C) Spectrophotometric activity assay of WT StSPL. The red curve represents the visible spectrum of the native protein before addition of substrate, corrected by the dilution factor. The black curves depict the visible spectra at regular intervals (1 min, 2, 4, 6, 8, 10, 12, 15, and 30 min) after addition of S1P. The transient peaks at 420 and 403 nm appearing upon addition of substrate correlate with protein activity. (D) Mass spectrometric activity assay of WT StSPL. The left panel depicts the reaction mixture measured just after mixing protein and substrate. The m/z 163.07 and 380.26 peaks correspond to the end product phosphoethanolamine and the substrate S1P, respectively. The right panel shows the reaction mixture after 75 min incubation at 20°C. No peak corresponding to S1P was detectable above background level.

The previously cloned full-length STH1274 gene was expressed in *E. coli* and the StSPL was purified to homogeneity as described [32]. The purity of the monomeric StSPL, which is a 507 amino acid protein with a calculated molecular weight of 55 kDa, was verified by SDS-PAGE followed by Coomassie staining of the gel (Fig. 1 A, lane 2) and Western blotting using an antibody recognizing the C-terminal His-tag (Fig. 1 A, lane 3). Based on our previous work which resolved the structure of WT StSPL at 2.0 Å resolution [32], we conclude that StSPL is a typical type I-fold dimeric pyridoxal 5′-phosphate (PLP)-dependent enzyme (Fig. 1 B) where residues from both subunits contribute to the active site. A phosphate ion coming from the buffer (red dot in Fig. 1 B) sits near the cofactor PLP (blue hexagon in Fig. 1 B) in the active site, mimicking the binding of the phosphate head of the substrate. The stretch of the StSPL chain spanning residues 1 to 57 (named Nt-FLEX) was not visible in the electron density map due to disorder. WT StSPL was shown to be active *in vitro* using two complementary activity assays (Figs. 1C and 1D). The first assay indirectly monitored the cleavage of the S1P substrate by recording spectrophotometric changes of the cofactor upon catalysis [32]. After addition of S1P to WT StSPL, the initial broad peak at 420–460 nm transiently disappeared and was replaced by a double peak at 420 & 403 nm (Fig. 1 C). The visible spectrum of the inactive mutant K311A or of an inhibited StSPL did not undergo any changes upon addition of substrate [32]. The second activity assay relies on mass spectrometry and monitors the disappearance of the S1P peak at m/z = 380.26 after incubation with StSPL (Fig. 1 D).

### StSPL is active under extracellular conditions

**Figure 2:**
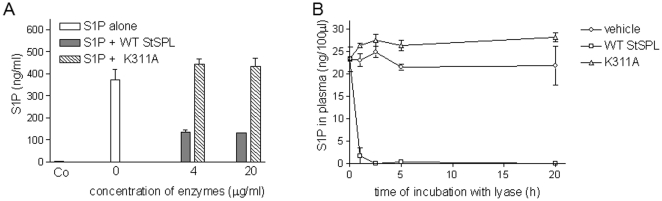
WT StSPL degrades S1P *in vitro*. (A) Medium (DMEM) was incubated for 30 min at 37°C with either vehicle (Co) or S1P in the absence (0, open bar ) or presence of the indicated concentrations of WT StSPL (StSPL; closed bars) or the K311A mutant (hatched bars). Thereafter, 100 µl of the medium was taken for lipid extraction and S1P was quantified by LC-MS/MS. Data are expressed as ng/ml of S1P and are means ± SD (n = 3). (B) Human plasma was incubated at 37°C for the indicated time periods (in hours) with either buffer (vehicle, circles), 20 µg/ml of WT StSPL (StSPL; squares), or 20 µg/ml of the K311A mutant (triangles). 100 µl plasma was taken for lipid extraction and S1P was quantified by LC-MS/MS. Data are expressed as ng/100 µl of S1P and are means ± SD (n = 3).

To investigate, whether StSPL is active also in the extracellular environment, the WT enzyme was added to cell culture medium supplemented with S1P and incubated at 37°C. As shown in Fig. 2A, S1P was degraded by 70% within 30 min, suggesting that even under extracellular conditions S1P is enzymatically degraded. In contrast, the K311A mutant of StSPL, which lacks the catalytically essential Schiff base bond with PLP, did not reduce medium S1P levels (Fig. 2A).

To see whether StSPL is also active in blood and capable of degrading blood-derived S1P, human plasma was prepared from healthy donors and incubated *in vitro* with WT StSPL or the K311A mutant. As shown in Fig. 2B, incubation of plasma at 37°C with buffer only did not alter the S1P level over a time period of 20 h. Moreover, there was no increase of sphingosine over 24 h of incubation (data not shown). These data demonstrate that S1P is rather stable in plasma depleted of blood cells, and excludes the spontaneous hydrolysis of S1P or an active degradation by other plasma factors such as plasma phosphatases. Incubation of plasma samples with WT StSPL rapidly degraded blood-derived S1P within 1 h of incubation, whereas control incubation with K311A did not affect S1P levels (Fig. 2B).

### StSPL disrupts S1P-stimulated proliferation and fibrotic response in renal mesangial cells

**Figure 3:**
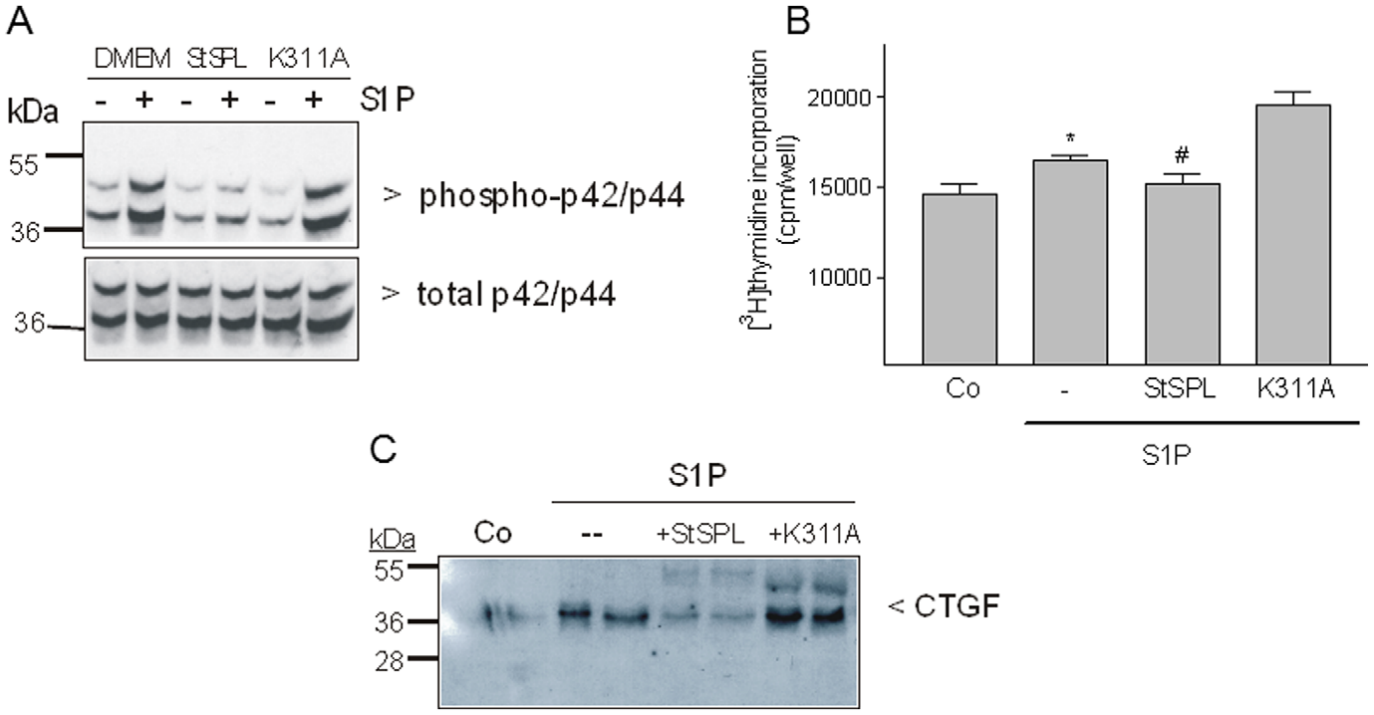
Effect of StSPL on S1P-stimulated MAPK phosphorylation, cell proliferation and CTGF expression in renal mesangial cells. (A) Quiescent rat mesangial cells were treated for 10 min with either vehicle (DMEM, -) or S1P (1 µM) in the absence or presence of WT StSPL (StSPL; 10 µg/ml) or the K311A mutant (10 µg/ml). Thereafter, cell lysates were separated by SDS-PAGE, transferred to nitrocellulose and subjected to Western blotting using antibodies against phospho-p42/p44 (dilution of 1∶1000, upper panel) and total p42/p44-MAPK (dilution each 1∶6000, lower panel). Blots were stained by the ECL method according to the manufacturer's recommendation. Data are representative of five independent experiments. (B) Quiescent cells were treated for 28 h with either vehicle (Co) or S1P (1 µM) which had been pretreated for 30 min at 37°C with either vehicle (-), WT StSPL (StSPL; 20 µg/ml) or the K311A mutant (20 µg/ml) in the presence of [^3^H]thymidine. Incorporated radioactivity was measured as described in the Materials and Methods section. Results are expressed as cpm/well of incorporated [^3^H]thymidine and are means ±S.D. (n = 4). (C) Quiescent cells were treated for 2 h as indicated above, and proteins were precipitated from the supernatants and taken for SDS-PAGE, transfer to nitrocellulose membranes and Western blotting using a CTGF-specific antibody (dilution 1∶1000). *p<0.05 considered statistically significant when compared to the vehicle-treated control values; ^#^p<0.05 when compared to the S1P-treated values (one-tailed p value).

To analyse the biological effects of StSPL on renal mesangial cells as an *in vitro* model mimicking glomerular fibrosis, we tested the activity of purified StSPL on intact cells and assessed its ability to interfere with S1P signalling. To this end, we first tested renal mesangial cells, since S1P-triggered responses are well defined in these cells. The stimulation of mesangial cells with S1P for 10 min resulted in an increased phosphorylation and thus activation of the classical p42- and p44-MAPK/ERKs (Fig. 3A, upper panel), which corroborates our previous finding (Xin et al., 2004). In the presence of WT StSPL, the S1P-triggered phosphorylation of p42- and p44-MAPKs was prevented, whereas the K311A mutant had no effect on the S1P-stimulated MAPKs (Fig. 3A).

S1P acts as a mitogen in renal mesangial cells [33], [34] and induces fibrosis as shown by upregulation of CTGF [35], [36], which represents a marker of fibrotic responses *in vivo* [37], [38]. Mesangial cell proliferation was measured by [^3^H]thymidine incorporation into de novo synthesized DNA. Treatment of quiescent mesangial cells with S1P for 28 h induced a moderate but significant increase in cell proliferation (Fig. 3B), which was prevented by WT StSPL but not the K311A mutant (Fig. 3B). Furthermore, we previously demonstrated that S1P activates gene transcription and de-novo protein synthesis of pro-fibrotic CTGF in mesangial cells [35]. As shown in Fig. 3C, this effect of S1P was also prevented by WT StSPL, but not K311A.

These data suggest that extracellular StSPL not only abolishes S1P-mediated effects on acute cellular signalling cascades, but also reduces S1P-triggered cell responses such as proliferation and fibrotic reactions in cell culture models.

### StSPL disrupts S1P-stimulated proliferation and migration of endothelial cells

**Figure 4:**
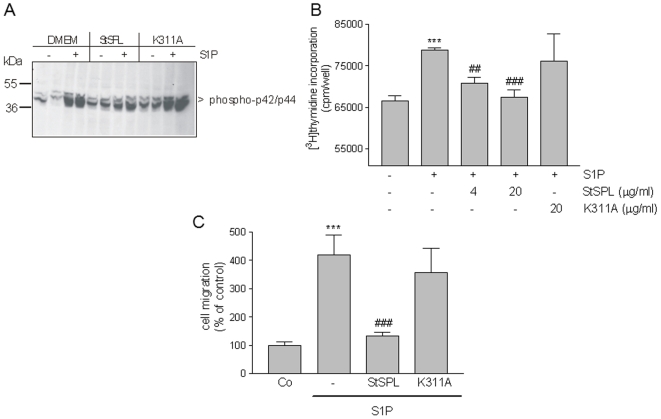
Effect of StSPL on S1P-stimulated MAPK phosphorylation, cell proliferation and migration of endothelial cells. (A) Quiescent EA.hy 926 human endothelial cells were treated for 10 min with either vehicle (Co) or S1P (1 µM) in the absence or presence of WT StSPL (StSPL; 10 µg/ml) or the K311A mutant (10 µg/ml). Cell lysates were prepared and separated by SDS-PAGE, transferred to nitrocellulose and subjected to Western blotting using antibodies against phospho-p42/p44 (dilution of 1∶1000, upper panel) and total p42/p44-MAPK (dilution each 1∶6000, lower panel). Data are representative of four independent experiments. (B) Quiescent cells were treated for 28 h with either vehicle (-) or S1P (1 µM), which had been pretreated for 30 min at 37°C with either vehicle (-), WT StSPL (StSPL) or the K311A mutant, in the presence of [^3^H]thymidine. Incorporated radioactivity was measured as described in the Materials and Methods section. Results are expressed as cpm/well of incorporated [^3^H]thymidine and are means ±S.D. (n = 4). ***p<0.001 considered statistically significant when compared to the vehicle treated control values; ^##^p<0.01, ^###^p<0.001 when compared to the S1P-treated values by one-way ANOVA analysis and Bonferroni post test. (C) Quiescent cells were treated for 14 h with DMEM (Co) or S1P (1 µM) which had been pretreated for 30 min at 37°C with either vehicle (-), WT StSPL (StSPL) or the K311A mutant. Thereafter, migrated cells were analysed as described in the Materials and Methods section. Results are expressed as migrated cells per counted field and are means ±S.D. (n = 3). ***p<0.001 considered statistically significant when compared to the vehicle treated control values; ^###^p<0.001 when compared to the S1P-treated values.

As an *in vitro* model of diseases associated with aberrant angiogenesis, the effect of StSPL on the human endothelial cell line EA.hy 926 was investigated. Again, S1P stimulated classical p42/p44-MAPKs phosphorylation, which was blocked by WT StSPL but not the K311A mutant (Fig. 4A).

In endothelial cells, S1P stimulates molecular events underlying angiogenesis, which includes cell proliferation and migration [39]. Indeed, we found that S1P stimulated EA.hy 926 cell proliferation (Fig. 4B), which was impeded by WT StSPL but not K311A (Fig. 4B). Moreover, undirected endothelial cell migration was also stimulated by S1P as measured in an adapted Boyden chamber assay (Fig. 4C), and this effect was similarly prevented by WT StSPL but not K311A (Fig. 4C). To exclude that the endothelial cells were stimulated by the degradation products of S1P, they were treated with 2E-hexadecenal at a concentration of up to 10 µM. However, we could not measure an effect on MAPK activation (data not shown). Our data demonstrate the potential of StSPL to combat aberrant angiogenesis commonly associated with diseases like cancer, diabetic retinopathy and macular degeneration.

### StSPL disrupts S1P-stimulated malignant responses in breast and colon carcinoma cells

**Figure 5:**
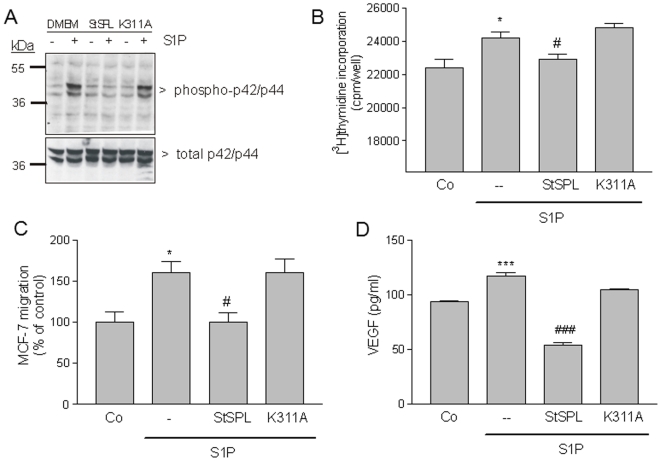
Effect of StSPL on S1P-stimulated MAPK phosphorylation, proliferation and migration and VEGF synthesis in MCF-7 breast carcinoma cells. (A) Quiescent MCF-7 cells were treated for 10 min with either vehicle (DMEM, -) or S1P (1 µM) in the absence or presence of WT StSPL (StSPL; 10 µg/ml) or the K311A mutant (10 µg/ml). Cell lysates were prepared and separated by SDS-PAGE, transferred to nitrocellulose and subjected to Western blotting using antibodies against phospho-p42/p44 (dilution of 1∶1000, upper panel) and total p42/p44-MAPK (dilution each 1∶6000, lower panel). (B) Quiescent MCF-7 cells were treated for 24 h with either vehicle (Co) or S1P (1 µM), which had been pretreated for 30 min at 37°C with either vehicle (-), WT StSPL (StSPL; 10 µg/ml) or the K311A mutant (10 µg/ml), in the presence of [^3^H]thymidine. Incorporated radioactivity was measured as described in the Materials and Methods section. Results are expressed as cpm/well of incorporated [^3^H]thymidine and are means ±S.D. (n = 4). (C) Quiescent MCF-7 cells were treated for 24 h with DMEM (Co) or S1P (1 µM), which had been pretreated for 30 min at 37°C with either vehicle (-), WT StSPL (StSPL) or the K311A mutant. Thereafter, migrated cells were analysed as described in the Materials and Methods section. Results are expressed as migrated cells per counted field and are means ±S.D. (n = 3). (D) Quiescent MCF-7 cells were treated for 24 h with DMEM (Co) or S1P (1 µM) which had been pretreated for 30 min at 37°C with either vehicle (-), WT StSPL (StSPL; 10 µg/ml), or the K311A mutant (10 µg/ml). Thereafter, supernatants were taken for a VEGF ELISA. Results are expressed as pg/ml of VEGF and are means ±S.D. (n = 4). *p<0.05, ***p<0.001 considered statistically significant when compared to the vehicle treated control values; ^#^p<0.05, ^###^p<0.001 when compared to the S1P-treated values.

**Figure 6:**
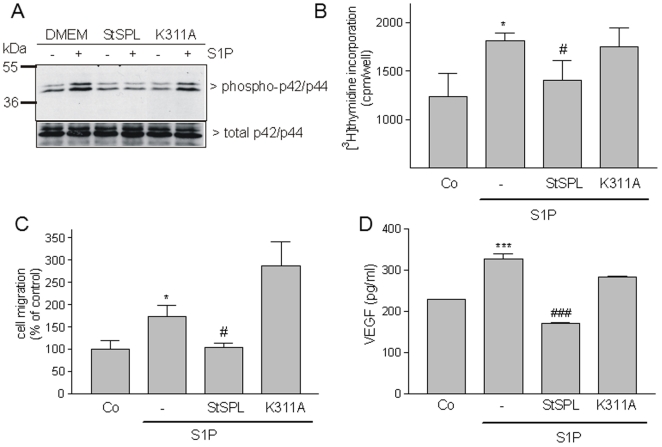
Effect of StSPL on S1P-stimulated MAPK phosphorylation, proliferation and migration and VEGF synthesis in HCT 116 colon carcinoma cells. (A) Quiescent HCT 116 cells were treated for 10 min with either vehicle (DMEM, -) or S1P (1 µM) in the absence or presence of WT StSPL (StSPL; 10 µg/ml) or the K311A mutant (10 µg/ml). Cell lysates were prepared and separated by SDS-PAGE, transferred to nitrocellulose and subjected to Western blotting using antibodies against phospho-p42/p44 (dilution of 1∶1000, upper panel) and total p42/p44-MAPK (dilution each 1∶6000, lower panel). (B) Quiescent HCT 116 cells were treated for 28 h with either vehicle (Co) or S1P (1 µM), which had been pretreated for 30 min at 37°C with either vehicle (-), WT StSPL (StSPL; 10 µg/ml) or the K311A mutant (10 µg/ml), in the presence of [^3^H]thymidine. Incorporated radioactivity was measured as described in the Materials and Methods section. Results are expressed as cpm/well of incorporated [^3^H]thymidine and are means ±S.D. (n = 4). (C) Quiescent HCT 116 cells were treated for 14 h with DMEM (Co) or S1P (1 µM), which had been pretreated for 30 min at 37°C with either vehicle (-), WT StSPL (StSPL) or the K311A mutant. Thereafter, migrated cells were analysed as described in the Materials and Methods section. Results are expressed as migrated cells per counted field and are means ±S.D. (n = 3). (D) Quiescent HCT-116 cells were treated for 14 h with DMEM (Co) or S1P (1 µM) which had been pretreated for 30 min at 37°C with either vehicle (-), WT StSPL (StSPL; 10 µg/ml), or the K311A mutant (10 µg/ml). Thereafter, supernatants were taken for a VEGF ELISA. Results are expressed as pg/ml of VEGF and are means ±S.D. (n = 4). *p<0.05, ***p<0.001 considered statistically significant when compared to the vehicle treated control values; ^#^p<0.05, ^###^p<0.001 when compared to the S1P-treated values.

There is convincing evidence that S1P contributes to tumorigenesis and malignant progression by promoting cell growth and metastasis [3]. Therefore, we investigated whether StSPL can also attenuate S1P-stimulated cell responses in tumor cells like the breast carcinoma cell line MCF-7 and the colon carcinoma cell line HCT 116. As shown in Fig. 5A and 6A, in both cell lines S1P stimulated classical p42/p44-MAPKs phosphorylation, which was prevented by WT StSPL but not the K311A mutant. Moreover, both cell lines responded to S1P stimulated by [^3^H]thymidine incorporation into DNA and this effect was again specifically impeded by WT StSPL (Fig. 5B and 6B). Similarly, S1P stimulated migration of HCT 116 (Fig. 5C) and MCF-7 (Fig. 6C) cells, and this effect was also impeded by WT StSPL. In addition to migration, WT StSPL drastically reduced S1P-stimulated VEGF secretion in HCT 116 (Fig. 5D) and MCF-7 (Fig. 6D) cells.

These findings demonstrate the ability of StSPL to effectively impede also the pro-malignant effect of S1P on carcinoma cells.

### StSPL is active *in vivo* and decreases plasma S1P levels in mice

**Figure 7:**
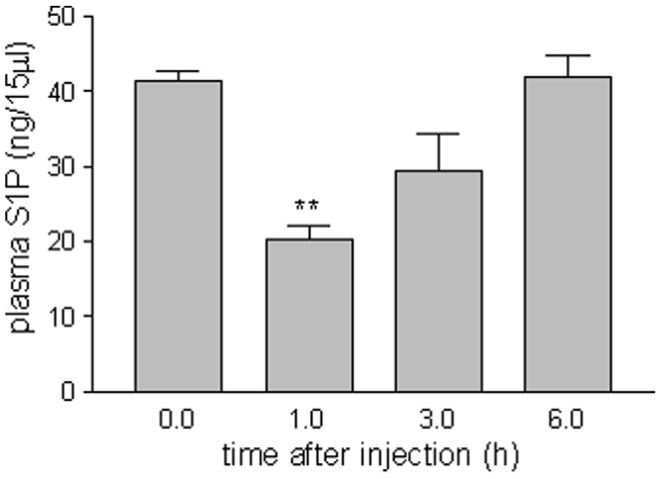
*In vivo* activity of intravenously injected StSPL in mice. WT StSPL (200 µg in 100 µl PBS per mouse) was injected intravenously into nude mice (n = 4). Blood aliquots were taken from a lateral tail vein either before injection (0) or after 1 h, 3 h, and 6 h. Plasma was prepared and taken for lipid extraction as described in the Materials and Methods section. S1P was quantified by LC/MS/MS as described. Data are expressed as ng/15 µl S1P and are means ±S.D. (n = 4). **p<0.01 considered statistically significant when compared to the control values by one-way ANOVA analysis and Bonferroni post test.

To investigate whether StSPL is also active under extracellular conditions *in vivo*, the WT enzyme was injected in mice and the degradation of S1P in mouse plasma was measured. As shown in Fig. 7, 1 h after injection of StSPL plasma S1P levels (determined as 40 ng in 15 ul) decreased to about 70%. After 3 h, S1P levels were partly recovered and normal control levels were reached 6 h after injection (Fig. 7). Although this indicates that there is scope for further pharmacological improvements to enhance efficacy, it clearly demonstrates that recombinant StSPL retains its enzymatic acitivity also *in vivo* upon intravenous injection. On the other hand, it indicates that the S1P blood pool was effectively replenished by continuous production in blood cells and that StSPL was eliminated from the circulation.

### StSPL inhibits tumor cell-induced angiogenesis in the chicken chorioallantoic membrane

**Figure 8:**
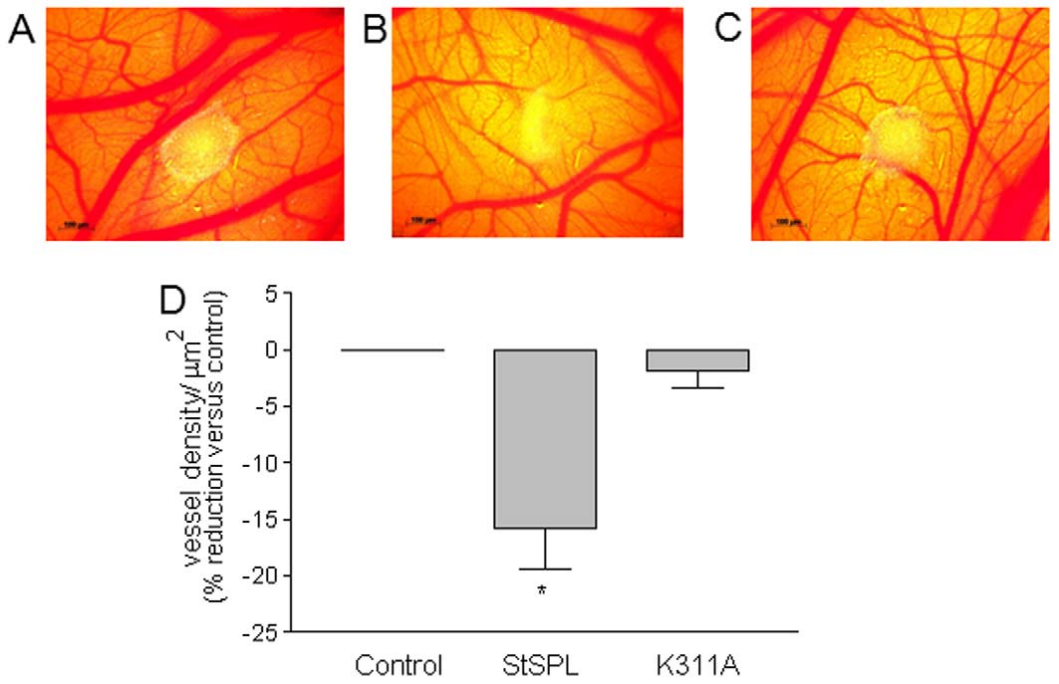
*In vivo* effect of StSPL on angiogenesis in the chicken chorioallantoic membrane (CAM). MCF-7 cell spheroids containing 5×10^5^ cells in 50 µl were placed on E8 CAMs, and either treated with PBS (control), WT StSPL (StSPL, 20 µg/ml), or K311A (20 µg/ml) for 4 d. CAMs were analysed for vessel formation as described in the Materials and Methods section, and the density of vessels per µm^2^ of area around the tumor was determined. Representative CAMs that were PBS-treated (A), WT StSPL-treated (B), and K311A mutant-treated (C) were photographed under a stereomicroscope and vessel density was determined using the Vessel_tracer software [58] (D). Results are expressed as vessel density per µm2 and are means +/− S.D. (n = 5). *p<0.05 considered statistically significant when compared to the control treated samples.

To demonstrate that StSPL can alter an S1P-dependent phenotype also under *in vivo* conditions, we investigated its effect on neovascularisation in the developing chorioallantoic membrane (CAM) of the chicken embryo. As a trigger for angiogenesis, spheroids of MCF-7 cells, which showed increased VEGF secretion upon StSPL treatment (Fig. 6D) were placed on the CAM of E8 chicken embryos. The ability of tumor cells including MCF-7 to induce vessel formation under these conditions [40] was described. As shown in Fig. 8, when MCF-7 cell spheroids were further incubated on the CAM for 4 d in the presence of StSPL, vessel formation was significantly decreased by 16% compared to PBS-treated CAMs. In contrast, vessel formation in the CAM was not affected by treatment with the inactive K311A mutant (Fig. 8).

## Discussion

In this study we describe for the first time the ability of recombinantly produced S*ymbiobacterium thermophilum* S1P lyase (StSPL) to degrade S1P *in vitro* and *in vivo*, thereby disrupting S1P receptor signaling in various disease models.

We have recently solved the structure of WT StSPL at 2.0 Å resolution [32]. The natural function of StSPL in the bacteria is still unknown but is most likely to catabolise various sphingolipids present in the environment. The first residues of StSPL align with the end of the predicted transmembrane helix of yeast and mammalian S1P lyases. StSPL lacks therefore an N-terminal transmembrane helix. In our previous study, we showed that in spite of this difference, N-terminal flexibility is a conserved feature of SPLs from both *Symbiobacterium thermophilum* and yeast [32]. The “invisible” Nt-FLEX domain of StSPL (residues 1–57) is a proline-rich and basic sequence able to adopt various conformations, and its positive charge at neutral pH may promote interaction with the negatively charged heads of phospholipids in membranes, and even penetrate bilayers, a known feature of proline-rich regions in proteins, and accommodate its hydrophobic substrate into the active site [41], [42]. Here we found that recombinantly expressed and purified StSPL is soluble in hydrophilic conditions and retains its enzymatic activity in cell culture medium and in blood. This was not necessarily expected, since the eukaryotic S1P lyase orthologues are integral membrane proteins.

Upon intravenous injection of StSPL in mice, levels of the S1P substrate initially declined but then recovered within 6 h. The transient nature of the degradation effect may have two reasons: 1) continuous production of endogenous S1P, *e.g.* by blood and vascular endothelial cells [6], [7], [8], [9], and 2) elimination of active enzyme by metabolic processes and excretion. Whether the lack of pyridoxal-5′-phosphate in the extracellular environment can destabilize the active conformation of the enzyme remains to be determined too. Altogether, it is obvious that further functional improvements of the enzyme are warranted to extend its half-life in the circulation, *e.g.* by surface polyethyleneglycol (PEG)ylation or coupling to other compounds designed to increase the size of this 110 kDa dimeric protein. Moreover, since S1P is a survival factor indispensable for the function of several vital tissues such as the immune and the cardiovascular system, targeted delivery and accumulation of StSPL specifically in diseased tissues might avoid side effects. This opens intriguing perspectives for S1P-targeted therapy using ligand-guided carrier systems [43], [44].

Recently, a molecular sponge approach has been described which is based on the use of a monoclonal antibody to absorb circulating S1P [28]. Although conceptually similar to our approach, we believe that the use of antibodies for simple reversible absorption of S1P in extracellular tissues may less efficiently compete with the continuous release of S1P from various sources than irreversibly depleting the circulating S1P pool as StSPL does. Nevertheless, the reported results from *in vitro* and *in vivo* use of the antibody in tumor models are impressive and confirm the strategic advantage of specific S1P targeting compared to the use of available small molecules as S1P receptor antagonists. For example, the sphingosine analogue FTY720 has been commonly used as an immunosuppressive agent to treat autoimmune diseases based on the role of the S1P_1_ receptor in lymphocyte trafficking. The *in vivo* phosphorylated form of FTY720 initially acts as an S1P receptor agonist, but subsequently adopts an indirect antagonistic function by promoting receptor downmodulation and thus resistance to signaling [21], [26], [27]. This ambivalent mode of action makes the effect of FTY720 *in vivo* rather unpredictable. Functionally improved receptor binding molecules including antagonistic antibodies are in preparation, but the low availability of properly folded purified G protein-coupled receptors required for the specific selection of binders has limited premature enthusiasm in the field.

Both the antibody and the StSPL approaches target S1P function on the level of cell surface receptor activation. S1P is produced by sphingosine kinases and acts as an intracellular modulator of the sphingosine rheostat to promote cell survival, proliferation and various other biological effects. Intracellular S1P lyase usually keeps the pool of free S1P in check, thereby controlling its pro survival function against the pro-apoptotic effects of sphingosine and ceramide in the rheostat [45], [46]. Accordingly, knockdown of intracellular S1P lyase in cancer cells was shown to disrupt apoptosis and results in chemoresistance by Bcl-2/Bcl-xL upregulation [47]. Elevated S1P is causative or at least contributory to various pathophysiologic disorders. Besides the disease models addressed in this study, increased levels of S1P were found in the blood of diabetic patients [48] and upregulated sphingosine kinase 1 (SK1) has been detected in phagocytes of patients with sepsis where it promotes excessive production of pro-inflammatory cytokines [49]. Consequently, targeted inhibition of SK1 to reduce intracellular S1P production was demonstrated to have therapeutic potential in various preclinical studies. However, as with almost all kinase inhibitors, the problem of insufficient specificity and off-target effects of SK1 inhibitors remains to be solved.

In addition, S1P can be secreted from cells to establish an extracellular pool in various tissues including blood, from where it binds to distinct S1P surface receptors in an auto- and paracrine fashion [2], [14], [16]. Although the two major S1P pools cannot be functionally dissected in terms of pathologic significance, there is ample evidence that disruption of S1P receptor signaling alone without tackling the intracellular S1P pool has significant therapeutic potential [16], [21], [22].

Using distinct cell types as defined *in vitro* models of cancer, fibrosis and aberrant angiogenesis, which are commonly associated with increased S1P receptor signaling, we report the ability of StSPL to disrupt the biological effects stimulated by extracellular S1P. In breast and colon carcinoma cells, targeted degradation of S1P prevented MAPK activation, proliferation, migration and VEGF production. The inhibitory effect of StSPL on angiogenesis observed in the chicken chorioallantoic membrane clearly demonstrates its ability to alter an important S1P-dependent phenotype also under *in vivo* conditions. Thus, similar to the S1P antibody approach [28] this demonstrates the enormous potential of S1P targeting for cancer therapy. Furthermore, we found that in renal mesangial cells StSPL disrupted S1P-induced MAPK activation, cell proliferation, and expression of CTGF, suggesting its use also for the treatment of fibrotic diseases [38], [50].

Nevertheless, it remains to be examined whether the degradation products of S1P, which inevitably appear in the plasma upon StSPL treatment, have an effect on vessel physiology. We found that 2E-hexadecenal, at up to 10 µM, did not alter MAPK signalling in the human endothelial cell line EA.hy 926 (data not shown). However, a previous study showed that in mouse embryonic carcinoma cell lines, various degradation products of S1P, including hexadecanal, palmitate, phosphoethanolamine, and ethanolamine, could trigger cell proliferation already at a concentration of 5 nM [51]. In contrast, Kumar et al. [52] showed that 2E-hexadecenal at higher concentrations of 25 µM and 50 µM triggered cytoskeletal reorganization and apoptosis of HEK293, NIH3T3, and HeLa cells likely due to increased oxidative stress.

In summary, we demonstrate that recombinant StSPL can be used to effectively degrade extracellular S1P *in vitro* and *in vivo*, and that it has therapeutic potential for pathologic conditions associated with elevated S1P. Further preclinical investigations and protein engineering to optimize the pharmacologic profile and *in vivo* efficacy of this enzyme therapeutic for clinical application are warranted.

## Materials and Methods

### Chemicals and materials

Secondary horseradish peroxidase-coupled IgGs, Hyperfilm MP and enhanced chemiluminescence reagents were from GE Health Care Systems (Glattbrugg, Switzerland). S1P, C17-S1P, C17-sphingosine, C17-ceramide and 2E-hexadecenal were from Avanti Polar (Alabaster, AL, US). The antibody against phospho-p42/p44-mitogen-activated protein kinase (MAPK) was from Cell Signaling (Frankfurt am Main, Germany), antibodies against glyceraldehyde-3-phosphate dehydrogenase (GAPDH) (V-18) and connective tissue growth factor (CTGF) (L-20) were from Santa Cruz Biotechnology (Heidelberg, Germany), the total p42- and p44-MAPK antibodies were generated as previously described [53]. The vascular endothelial growth factor (VEGF) enzyme-linked immunosorbent assay (ELISA) was from R&D Systems Europe Ltd. (Abingdon, U.K.). All cell culture additives were from Invitrogen AG (Basel, Switzerland).

### Expression of recombinant WT StSPL and the K311A mutant in *E.coli*

The recombinant WT StSPL and the K311A mutant lacking the PLP binding residue were expressed in *E. coli*, purified in PBS containing 10 µM of PLP, and thoroughly structurally and functionally characterized as described [32]. The *in vitro* activity of StSPL was monitored using a spectrophotometric and a mass spectrometric activity assay. The first one indirectly monitors the cleavage of S1P while the second one directly records the cleavage of S1P [32].

### Cell culture

Rat renal mesangial cells were isolated and characterized as previously described [53]. The human endothelial cell line EA.hy 926 was obtained from Dr. Edgell (Chapel Hill, NC, USA) and cultured as previously described [54]. MCF-7 breast carcinoma and HCT 116 colon carcinoma cells were obtained from ATCC (American Type Culture Collection). MCF-7 cells were cultured in Dulbecco's modified Eagle medium (DMEM) containing 10% (v/v) fetal bovine serum, 6 µg/ml insulin, 100 units/ml penicillin and 100 µg/ml streptomycin, HCT 116 cells were cultured in McCoy medium containing 10% (v/v) fetal bovine serum, 100 units/ml penicillin and 100 µg/ml streptomycin. Prior to S1P stimulation, cells were rendered quiescent for 24 h in DMEM (for carcinoma cells phenolred-free medium was used) including 0.1 mg/ml of fatty acid-free bovine serum albumin (BSA).

### Western blotting

Stimulated cells were homogenised in lysis buffer and centrifuged for 10 min at 14000× g. The supernatant was taken for protein determination. 30 µg of protein were separated by sodium dodecylsulfate-polyacrylamide gel electrophoresis (SDS-PAGE), transferred to nitrocellulose membrane and subjected to Western blotting as previously described [55] using antibodies as indicated in the figure legends. For the detection of secreted CTGF, equal volumes of supernatants of stimulated cells were taken and proteins were precipitated with 7% trichloroacetic acid.

### Quantification of S1P by liquid chromatography/tandem mass spectrometry (LC/MS/MS)

15 µl of plasma samples or 100 µl of medium were taken for lipid extraction according to Bligh and Dyer [56], and lipids were quantified by liquid chromatography-coupled tandem mass spectrometry (LC/MS/MS) as described [57].

### [^3^H]Thymidine incorporation into DNA

Confluent cells were starved for 24 h in serum-free DMEM containing 0.1 mg/ml of BSA. Thereafter, cells were stimulated in the presence of [^3^H]methyl-thymidine (1 µCi/ml) in the absence or presence of S1P, and StSPL was added for further 24–28 h. Cells were processed as described [55].

### Cell migration assay

To measure undirected cell migration, an adapted Boyden chamber assay was performed as described [54].

### Quantification of VEGF

Secretion of vascular endothelial growth factor (VEGF) into cell culture medium was quantified by ELISA (R&D Systems Europe Ltd., Abingdon, U.K.) as recommended by the manufacturer. Confluent cells in 24-well-plates were stimulated in a volume of 0.5 ml.

### *In vivo* activity of StSPL

Experiments were approved by the commission for animal experimentations of the Veterinäramt of the Kanton Bern (approval No. 43/09). 10 week old female CD1 mice (Charles River, Sulzfeld, Germany) were injected intravenously with 200 µg WT StSPL in 100 µl phosphate-buffered saline (PBS). Blood was taken either before treatment (control) or 1 h, 3 h and 6 h after injection by collecting 100 µl blood from the lateral tail vein using a heparinised capillary. Samples were centrifuged for 10 min at 2000× g and the supernatant (plasma) was taken for further quantification of S1P by LC/MS/MS.

### Chicken chorioallantoic membrane (CAM) model of angiogenesis

A shell-free culture method was used to obtain chorioallantoic membranes (CAM) of chicken embryos. Fertilized chicken eggs (Brüterei E. Wüthrich AG, Belp, Switzerland) at embryonic day 4 (E4) were opened and placed into plastic dishes (Thermoflex AG, Switzerland) and further incubated at 37°C and 55% relative humidity. At E8, 5×10^5^ MCF-7 cell spheroids, which were prepared in growth medium containing 0.2% methylcellulose, were placed on the CAM and treated with either PBS, StSPL, or the K311A mutant as indicated in the figure legend. At E12, CAMs were examined for vessel formation under a stereomicroscope (Carl Zeiss AG, Feldbach, Switzerland). The density of vessels per area around the tumor was determined using the free downloadable software Vessel_tracer developed by Sofka and Stewart [58] (http://www.cs.rpi.edu/~sofka/vessels_exec.html).

### Statistical analysis

Statistical analysis was performed by using unpaired t-tests and one-tailed or two-tailed p values when comparing two groups, or by using one-way ANOVA analysis and Bonferroni post test when comparing more than two groups.
